# *In vitro* effects of hydroxyapatite containing toothpastes on dentin permeability after multiple applications and ageing

**DOI:** 10.1038/s41598-018-22764-1

**Published:** 2018-03-20

**Authors:** Karl-Anton Hiller, Wolfgang Buchalla, Isabel Grillmeier, Christina Neubauer, Gottfried Schmalz

**Affiliations:** 1 Department of Conservative Dentistry and Periodontology, University of Regensburg Medical Center, Regensburg, Germany; 2 Department of Periodontology, School of Dental Medicine, Bern, Switzerland; 3 Present Address: Private Practice, Nürnberg, Germany; 4 Present Address: Private Practices, Riedenburg and Regensburg, Germany

## Abstract

This *in vitro* study evaluated the effect of toothpastes with different active ingredients on dentin permeability using an extended protocol including multiple applications and several thermal ageing cycles in the presence or absence of human saliva. The Null hypothesis was that dentin permeability of a hydroxyapatite containing toothpaste (BR), a potassium nitrate (SP) and an arginine and calcium carbonate (EH) containing toothpaste were similar. Dentin permeability was measured as hydraulic conductance using a commercially available capillary flow system (Flodec, Geneva) and results were expressed as % relative to matching controls. Without saliva, the ranking (best first) of dentin permeability was BR(61%) < SP(87%) < EH(118%), with saliva EH(63%) < SP(72%) < BR(88%). Saliva increased or decreased permeability dependent upon the test material. BR reduced dentin permeability significantly more in absence of saliva, with saliva EH was superior to BR. Repeated material application decreased and thermal ageing increased dentin permeability. The different tooth pastes reduced permeability differently, the best being BR without saliva, the least EH without saliva. The newly introduced test conditions (ageing, saliva, multiple applications) influenced single results significantly, and as they better simulate the *in vivo* situation they should be considered to be included in further *in vitro* permeability testing of desensitizing preparations.

## Introduction

Toothache can have many reasons. In some cases, patients complain about pain after exposure of their teeth to cold or hot diet. This is a frequent symptom of a so-called dentin hypersensitivity (DH). Dentin contains about 30,000 tubuli per mm² of 1–2 µm diameter running perpendicularly from the dentin surface to the dental pulp^1^. In clinical dentistry DH is defined as a dentinal pain, which is sharp in character and of short duration, arising from exposed dentin surfaces in response to stimuli, typically thermal, evaporative, tactile, osmotic, chemical or electrical; and which cannot be ascribed to any other dental causes^2–6^. The prevalence rates of DH range largely between 3 and 98% depending on selection criteria, diagnostic approaches or time frames examined^7,8^. Exposure of dentin, seems to be a predisposing factor and is the consequence e.g. of abrasion, periodontal disease or enamel erosive wear^9–12^. Abfraction and vigorous tooth brushing were discussed as further possible etiological factors for DH^7^. Some authors claim that occlusal overload may lead to such symptoms, although scientific evidence is weak^13^. DH was found to be related to substantially impaired oral health related quality of life, when a patient population with this condition was compared with the general population^14^. Perception of pain may vary form slight discomfort to severely impaired quality of life^14,15^.

Generally, treatments for DH comprise nerve desensitization (e.g. by potassium nitrate), protein precipitation (e.g. by glutaraldehyde, silver nitrate, zinc chloride), blocking of dentinal tubules (see below), sealing by dentinal adhesives, or by laser application (Nd:YAG, GaAlAs, Er:YAG)^4,5,16–21^. In case of persisting discomfort, composite resin restorations (mainly Class V) or even endodontic treatment may become necessary^22^.

Treatment modalities for DH aimed at decreasing dentin permeability by blocking dentinal tubules are widely used and respective preparations are available for at home use, e.g. toothpastes, mouthwashes, or chewing gums^23–25^. Respective desensitizing toothpastes may contain e.g. hydroxyapatite, strontium chloride, strontium acetate, arginine with calcium carbonate, and calcium sodium phosphosilicate (“bioglass”) as active ingredients^26–28^. High concentrations of F^**-**^ leads to formation of CaF_2_ precipitates^29^.

It has been postulated that Arginine might interact with calcium carbonate particles which lead to a positively charged particle surface. These particles can bind to the negatively charged dentin and occlude open dentin tubuli. Additionally, the alkaline pH-value favors calcium phosphate precipitation from saliva^30,31^.

Synthetic hydroxyapatite (HAP) has been described as a biocompatible and biomimetic compound which is able to reduce hypersensitivity and to potentially enhance remineralization^18,19,32,33^. Recently, toothpastes containing HAP have been marketed and found to decrease DH efficiently clinically^34–37^.

A particular crystalline zinc substituted hydroxyapatite used for the treatment of DH was analyzed with various physicochemical methods. Because of its particle size of about 200–400 nm, these hydroxyapatite crystallites are well suited to occlude open dentin tubuli^24^.

For testing dentin permeability, different *in vitro* methods have been described, based either on measuring the diffusion or the perfusion of marker substances through dentin before and after material application^1,38–41^, simulating a one-time use of a product or compound. Thus, in all these *in vitro* test methods used so far, the situation in the oral cavity has only been simulated insufficiently. Toothpastes or other respective oral hygiene products are used regularly and repeatedly, sometimes more than once a day. Furthermore, assuming that the desensitizing effect is based on a mechanical blocking of the orifices of exposed dentin tubules, this blockage is subject e.g. to thermal stress in the oral cavity. Moreover, saliva apparently plays a role in reducing DH symptoms. According to Cummins^42,43^ arginine, especially together with calcium carbonate in saliva, has been made responsible for this effect. A desensitizing toothpaste has recently been marketed containing these substances aiming to enhance the effect of saliva. Also, hydroxyapatite as such interacts with saliva proteins forming the enamel pellicle due to its high polarity. Many different salivary proteins can bind to the enamel surface, e.g. proline-rich proteins, cystatins, statherins, histatins, and others. Two mechanisms of protein adsorption are reasonable, a direct mineral-protein interaction and a protein-protein interaction^44–48^.

Therefore, the aim of the present *in vitro* study was to evaluate the effect of toothpastes with different active ingredients on dentin permeability using an extended test protocol including multiple applications and several thermal ageing cycles in the presence or absence of human saliva. The Null hypothesis was that the dentin permeability of a HAP containing toothpaste (BR), a potassium nitrate (SP) and an arginine and calcium carbonate (EH) containing toothpaste were similar.

## Results

### Controls

**Figure 1:**
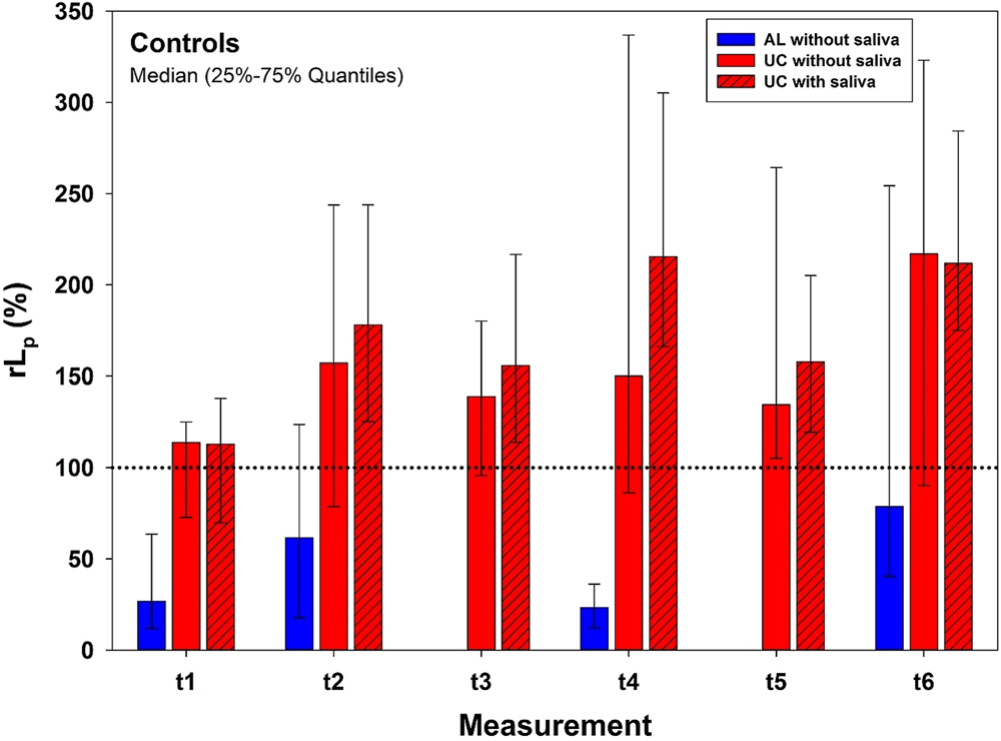
Results for controls. Relative Hydraulic conductance *rL*_*p*_ (%) for controls tested, without (for UC and AL) and with (for UC only) saliva for 6 measurement points. Dotted line represents baseline (t_0_) measurements which were set to 100% for each single dentin slice. AL was not measured at t_3_ and t_5_. Depicted are Medians and 25–75% Quantiles.

Hydraulic conductance *L*_*p*_ before treatment at baseline (t_0_) ranged (median) between 0.13 and 0.55e-10 m^3^N^−1^s^−1^ and was set to 100%. The relative hydraulic conductance *rL*_*p*_ of control AL, tested without saliva only, ranged between 23% and 79% (Fig. 1), being significantly below 100% (baseline) at all measurement points t_1_, t_2_, t_4_, and t_6_. AL was applied only once before t_1_ and measurements at t_3_ and t_5_, at which otherwise test materials were re-applied, were not performed. Relative hydraulic conductance *rL*_*p*_ of untreated control slices (UC) increased with time from 114% to 217% without saliva, and from 113% to 215% with saliva, respectively (Fig. 1). Without saliva, *rL*_*p*_ at t_5_ was significantly different from baseline, whereas with saliva, *rL*_*p*_ at all measurement time points except t_1_ were significantly higher compared to t_0_. Comparing *rL*_*p*_ of untreated controls with and without saliva, for t_2_, t_3_, t_4_ and t_5_ the median *rL*_*p*_ is higher for dentin slices coated with saliva compared to those without. However, in general and over all (Error rates method) the influence of saliva on *rL*_p_ could be verified only as a trend.

### Test materials

#### Overview

**Figure 2:**
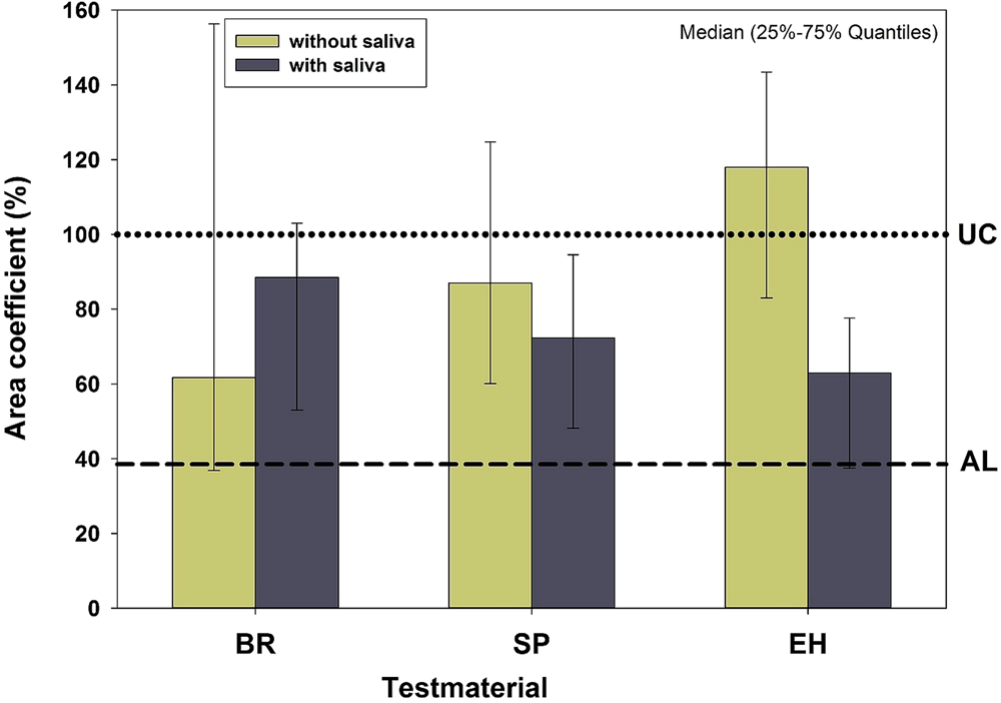
Overview of the results. *Area coefficients* (%) of materials tested without and with saliva. To gain an overview of the results, for each dentin slice the area under its *rL*_*p*_ curve (abszissa: measurement time; ordinate: corresponding *rL*_*p*_ value) was calculated, related to matching areas under the curves of the untreated controls (UC), and expressed as % (*area coefficient*; 100% ~ area under the curve of untreated control slices). Dotted and dashed lines depict the area coefficients of untreated controls (set to 100%) and AL, respectively. Depicted are Medians and 25–75% Quantiles.

Without saliva, the (best first) permeability ranking in terms of the *area coefficient* was BR (61%) < SP (87%) < EH (118%), whereas with saliva the ranking was EH (63%) < SP (72%) < BR (88%) (Fig. 2). Area coefficients of EH were significantly different with saliva compared to without saliva, and EH with saliva was significantly different to untreated control UC.

#### Influence of single materials

**Figure 3:**
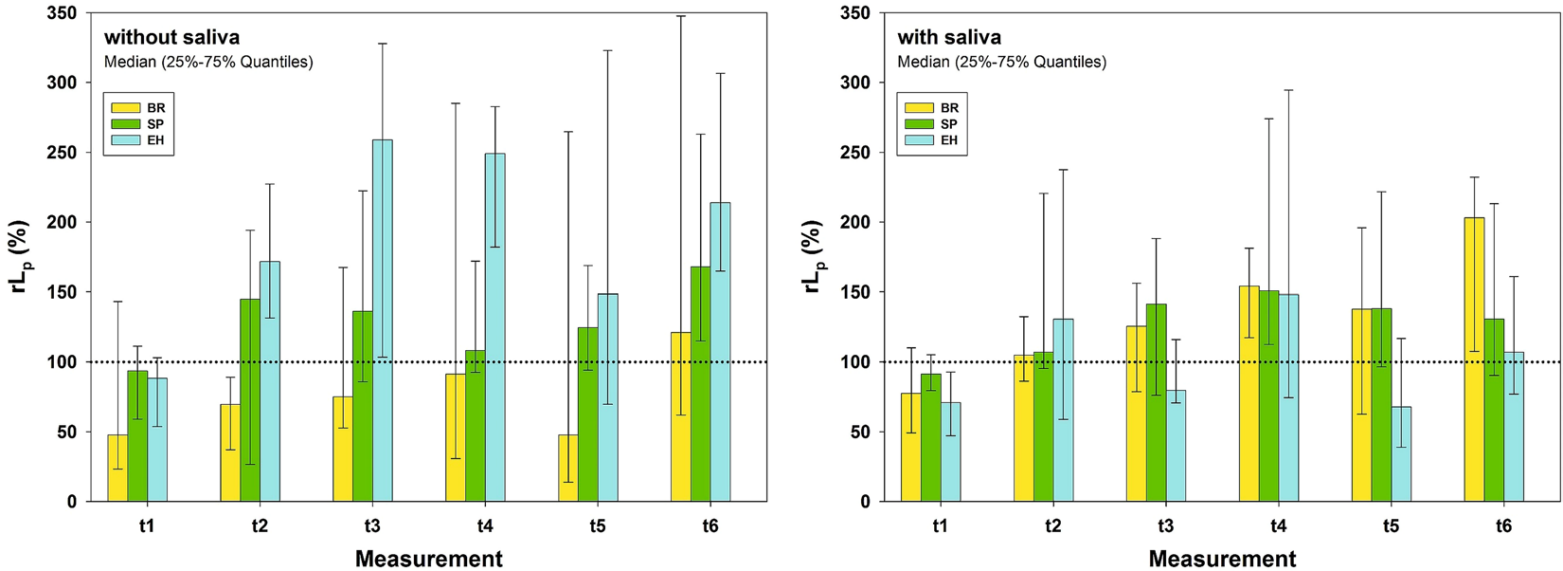
Results (*rL*_*p*_) related to time points. Relative Hydraulic conductance *rL*_*p*_ (%) for materials tested, without (left) and with (right) saliva for six measurement points. Baseline hydraulic conductance was set to 100% and visualized as dotted horizontal lines. Depicted are Medians and 25–75% Quantiles.

Results (*rL*_*p*_) for the test materials are depicted in Fig. 3, visualizing the baseline references (set to 100%) as dotted lines. Without saliva BR by tendency reduced *rL*_*p*_ over time compared to t_0_, (baseline) being significant at t_2_ (70%). Other test materials tended to increase *rL*_*p*_ over time up to 259% compared to t_0_, being significantly different from baseline in cases when the 25–75% quantiles interval did not include the 100% (dotted line) value. BR decreased significantly *rL*_*p*_ compared to EH (74% vs. 259%) at t_3_. At t_4_ SP (108%) was significantly different from EH (250%) (Fig. 3). The untreated control UC was significantly different from BR at t_2_ (Figs 1 and 3). With saliva EH generally tended to decrease *rL*_*p*_, being significantly lower at t_1_ (71% %) and t_5_ (68%) compared to t_0_ (100%). Other test materials tended to increase *rL*_*p*_ over time up to 203% compared to t_0_, being significantly different from baseline in cases when the 25–75% quantiles interval did not include the 100% (dotted line) value (Fig. 3). The untreated control UC was significantly different from BR at t_2_ and t_4_, and from EH at t_3_, t_5_ and t_6_ (Figs 1 and 3).

#### Influence of saliva

As already shown in Fig. 2 (overview), saliva in general only showed a tendency to influence the dentin permeability results. In detail, however, *rL*_*p*_ of EH at t_3_ and t_6_ was significantly different without saliva (259% and 214%, respectively) compared to with saliva (80% and 107%, respectively). Without saliva, BR showed by tendency lowest *rL*_*p*_ values compared to other test materials, whereas with saliva EH showed lowest *rL*_*p*_ values, although these were not statistically different in either situation (Fig. 3).

#### Influence of repeated material application and thermal ageing

**Figure 4:**
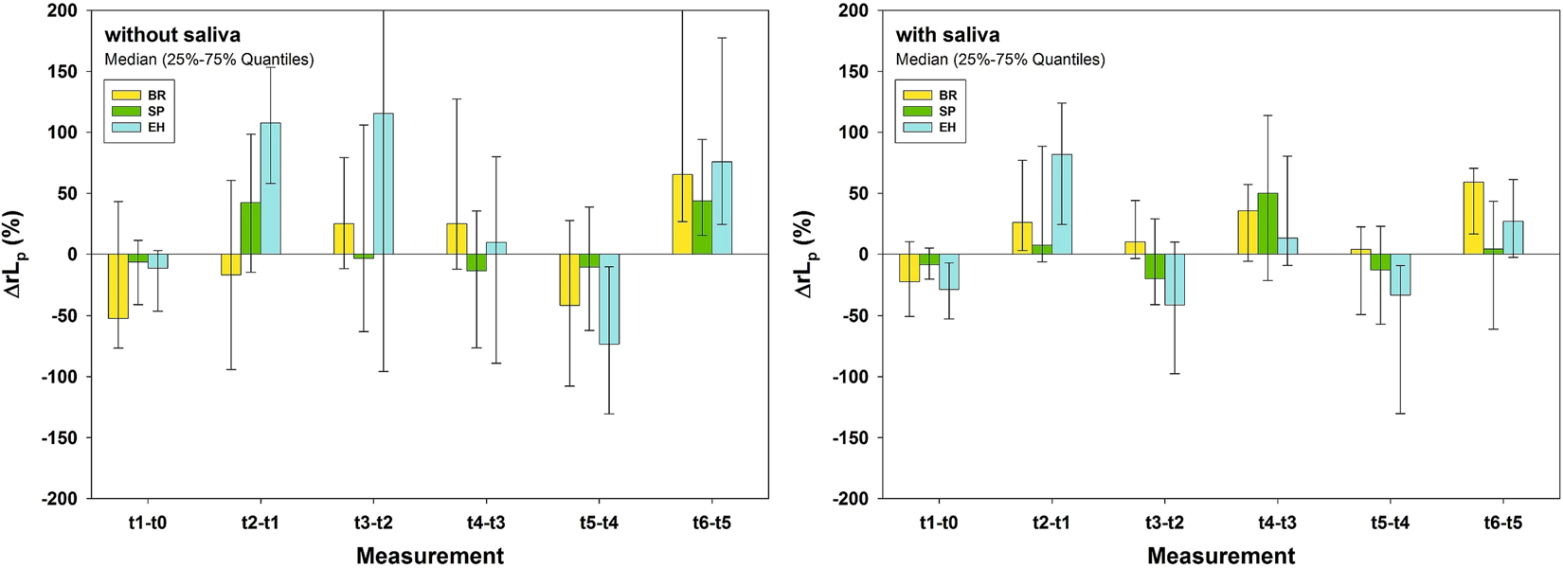
Results (*ΔrL*_*p*_) related to time differences. Successive differences of relative Hydraulic conductance *ΔrL*_*p*_ (%) for materials tested, without (left) and with (right) saliva for six measurement intervals. Depicted are Medians and 25–75% Quantiles.

Considering time differences, material application (t_1_-t_0_, t_3_-t_2_, t_5_-t_4_) tended to decrease *ΔrL*_*p*_, whereas thermal ageing (t_2_-t_1_, t_4_-t_3_, t_6_-t_5_) increased it for all test materials irrespective of presence or absence of saliva, though being more consistent with saliva (Fig. 4). For each material and time difference *ΔrL*_*p*_ without saliva was not significantly different compared to with saliva. Therefore, an overall influence of saliva on *ΔrL*_*p*_ could not be detected (Fig. 4).

#### SEM visualization

**Figure 5:**
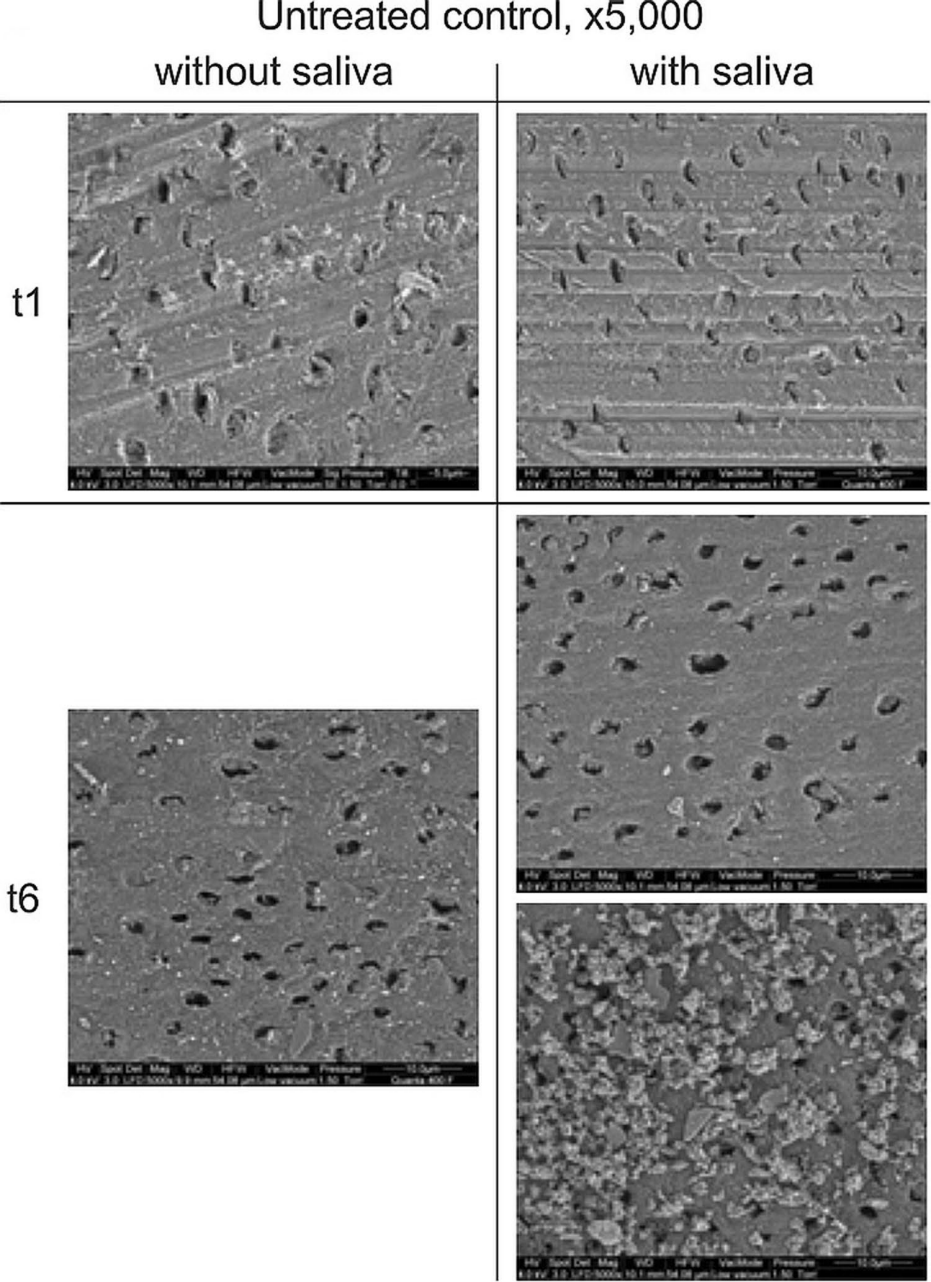
SEM, surface view of untreated controls. SEM visualization of the dentin surface of untreated controls (UC) before (t_1_) and after third thermal ageing period (t_6_), without and with saliva application at original magnifications of x 5,000 under environmental conditions.

SEM pictures from untreated controls with and without saliva show dentin tubuli before thermal ageing (t_1_), which are partially covered with dentin remnants from the grinding process. After the third thermal ageing period (t_6_) the dentin tubuli are mainly devoid of such remnants. However, in some of the samples with saliva a coating on the dentin surface was observed, apparently without occluding open orifices (Fig. 5).

**Figure 6:**
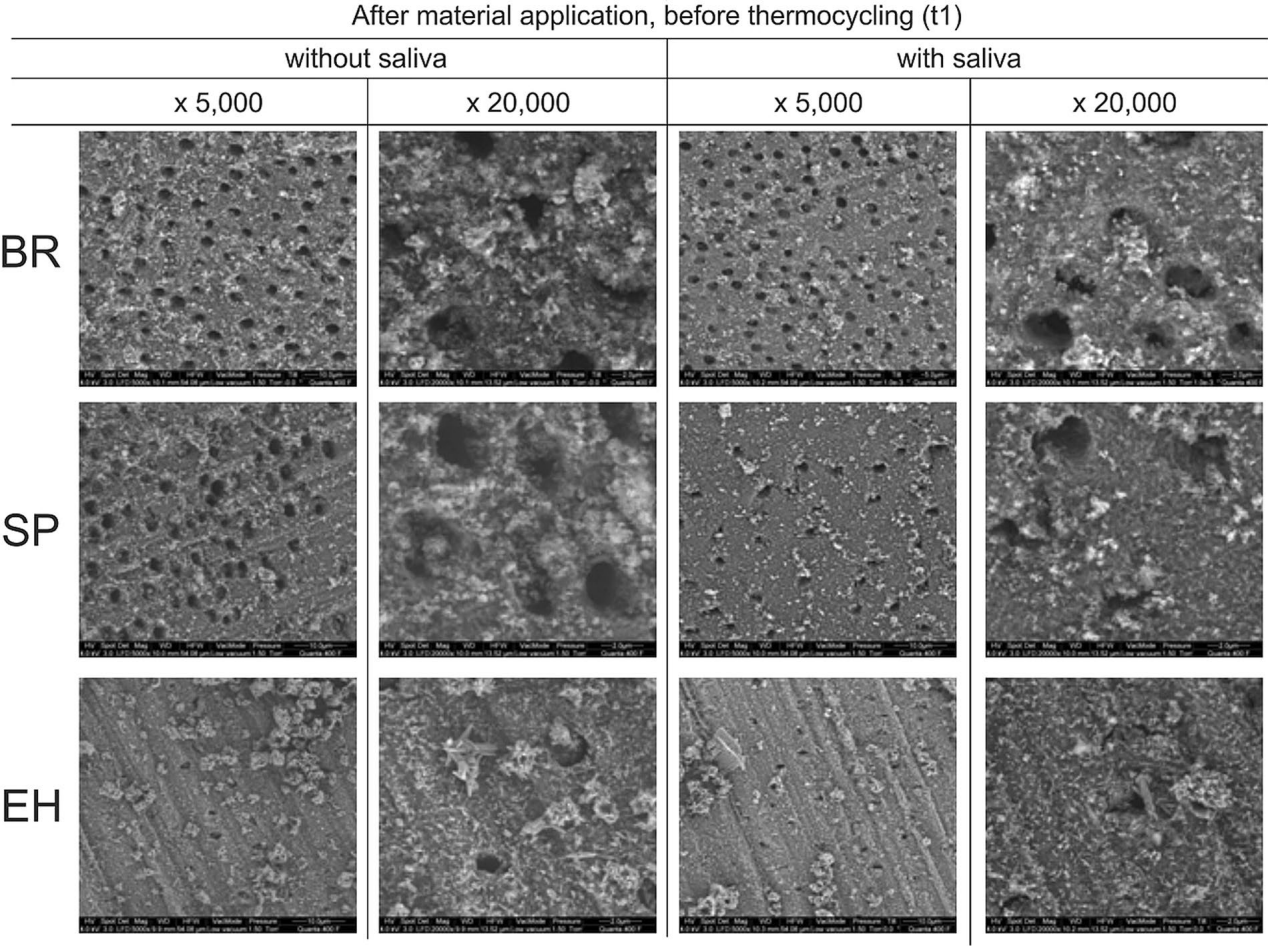
SEM, surface view at t_1_. SEM visualization of the dentin surface after first material application and before first thermal ageing (t_1_) for toothpastes containing hydroxyapatite (BR), potassium nitrate (SP), and calcium carbonate and arginine (EH), without and with saliva at original magnifications of x5,000 and x20,000 under environmental conditions.

After application of the test materials and before thermal ageing (t_1_) all materials produced a layer of deposits on the dentin surfaces. However, compared to other materials, EH shows a different morphology of these deposits with spindle-like crystals be apparent in the samples without saliva. There is a tendency that occlusion of tubules is more pronounced for EH with saliva compared to without saliva (Fig. 6).

**Figure 7:**
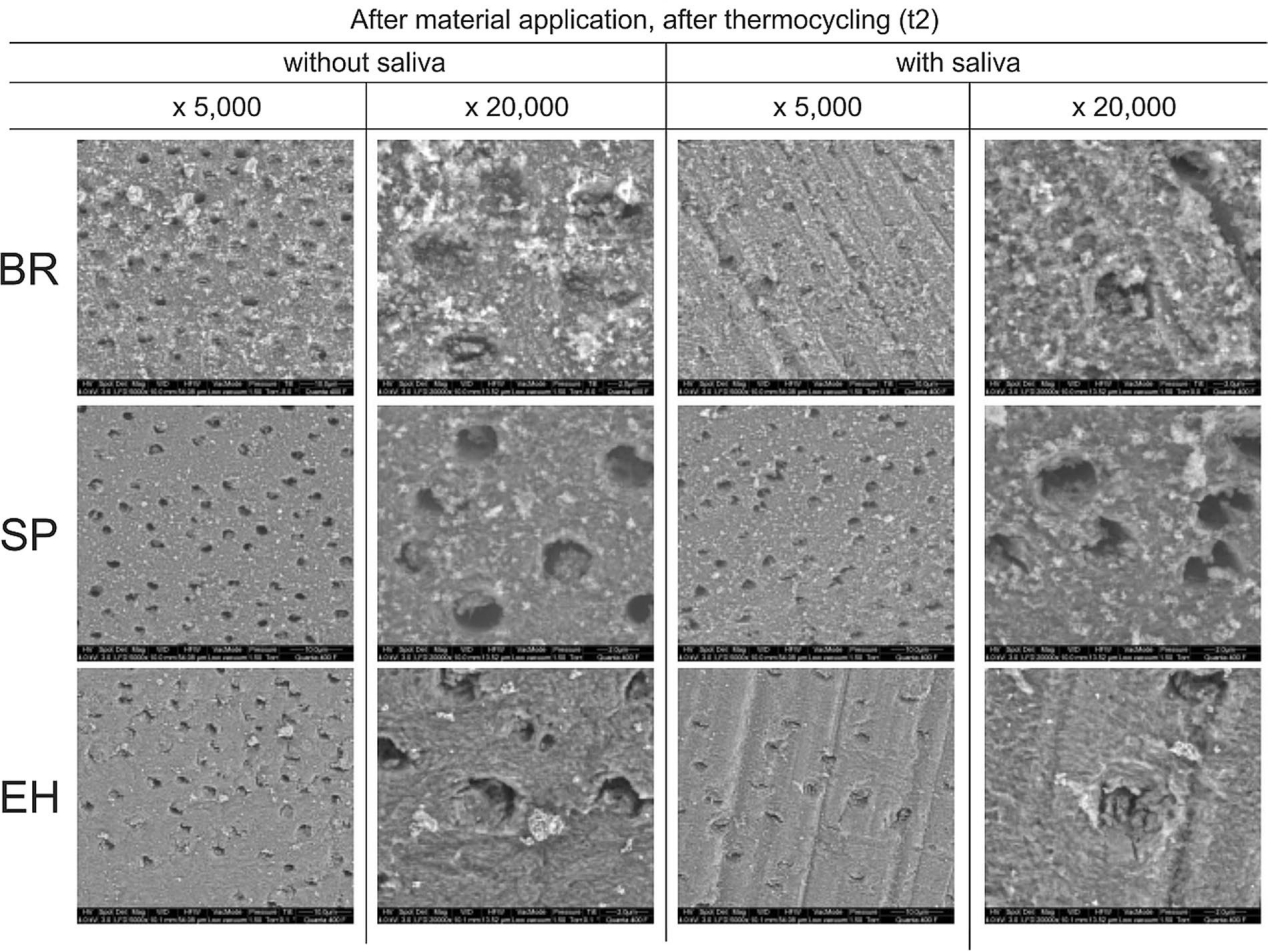
SEM, surface view at t_2_. SEM visualization of the dentin surface after first material application and after first thermal ageing period (t_2_) for toothpastes containing hydroxyapatite (BR), potassium nitrate (SP), and calcium carbonate and arginine (EH), without and with saliva, at original magnifications of x 5,000 and x 20,000 under environmental conditions.

After application of the test materials and after the first thermal ageing period (t_2_) for all materials the amount of deposit seems to be reduced compared to before thermal ageing (t_1_), although for EH with saliva again, there seems to be a tendency to have more occluded orifices compared to other materials. Without saliva, occlusion of tubules with BR seems to be more pronounced than for other preparations (Figs 6 and 7).

## Discussion

**Figure 8:**
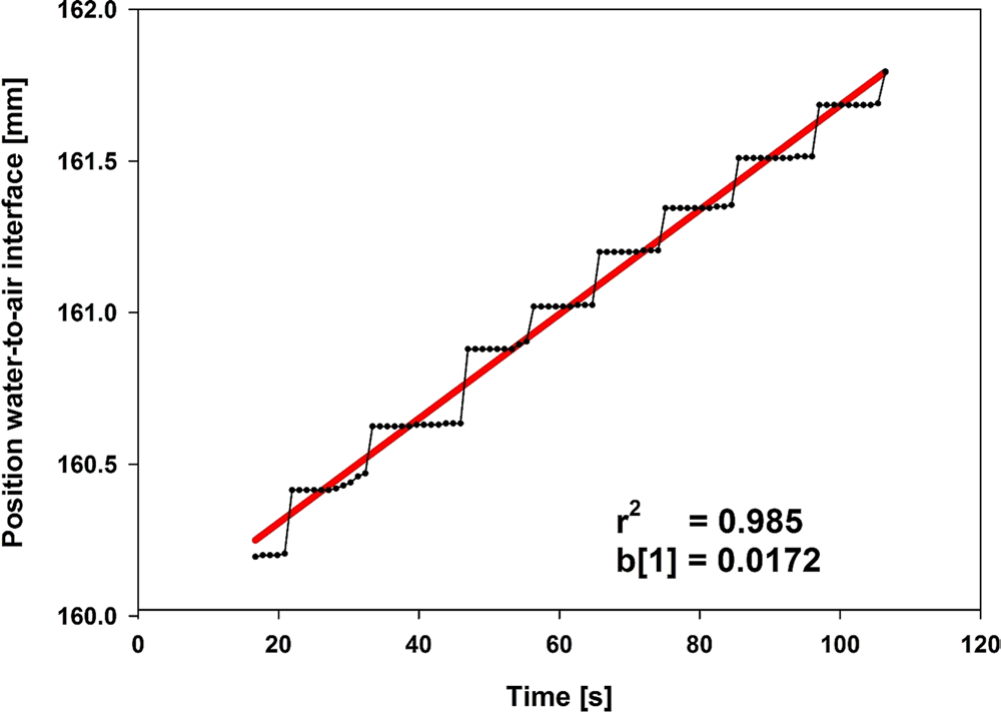
Data treatment. Printout of original data (recorded by the Flodec software; dotted) and the corresponding linear fit (red line) for calculation of hydraulic conductance (*L*_*p*_) with resulting slope b[1] and correlation coefficient r^2^.

Hydraulic conductance of untreated dentin slices in the present study ranged between 0.13 and 0.55e-10 m^3^N^−1^s^−1^, which is in line with published data of about 0.68 e-10 m^3^N^−1^s^−1^^41^. The upper value in the present study was 4.2 fold higher than the lower one, applying a constant pressure of 13.8 kPa. Generally, a major methodical problem of testing dentin permeability by measuring *hydraulic conductance* is the large variation among different dentin slices. To address this, we standardized the duration of the measurements to 2 min and discarded 15 s each from the beginning and the end to avoid any handling influences. The Flodec system, used frequently in the literature^49,50^, continuously recorded a fluid flow through the dentin slices by measuring the movement of a water-to-air interface within an internal circular glass capillary of constant diameter (according to manufacturers’ information). The pressure applied (1.4 m H_2_O = 13.8 kPa) was within the range (1.38–34.47 kPa) reported in the literature^40,49^. Applying this relatively low pressure, the generated time-traverse diagrams show a stepwise shape (Fig. 8). This may be due to the stepping motor of the sensor used, which is able to minimally resolve about 7 nl fluid change through the capillary inserted. The stepwise shape, containing several parts with no recorded additional movement during several seconds (Fig. 8) also indicates that “very short” measurement periods or even discontinuous measurements may lead to incorrect results^50^. We decided not to include longer measurement periods which are sometimes reported, e.g. 3 min^49^ or even 15 min^50^, because this would not provide additional information in our system, since we included a preselection criterion in the test protocol. In the present study, dentin slices were preselected according to the quality of their permeability characteristics. The quality of each single measurement was assessed in terms of the correlation coefficient r^2^ of the fit of the time-to-distance graphs recorded by the Flodec system (Fig. 8). A dentin slice was discarded, if just one of the seven correlation coefficients was below 0.7. The application of this relatively strong preselection criterion resulted in the exclusion of about half of the dentin slices (data not shown). As reported earlier, the permeability characteristics of bovine and human dentin are similar and the use of bovine instead of human dentin also contributed to a reduction of the variations^1^. To simulate the *in vivo* situation, dentin slices were etched on the pulp-facing side to remove the smear layer and to open the tubuli. The other side (treatment side) was ground to generate a standardized smear layer for all slices.

In order to compensate for large variations of the permeability measurements, an alternative approach has been described recently, where a constant flow rate of 15 µl/min was established to standardize the baseline situation before the measurements. This flow rate was achieved by varying the applied pressure in the range of 1.38 to 34.47 kPa^40^, the higher value being about 25 fold higher than the lower one.

In the present study and in this alternative approach^40^, consecutive measurements of a dentin slice were related to the matching baseline situation. By this method, each dentin slice serves as its own control, thereby also compensating for quite large variations among different dentin slices.

Toothpastes usually are used more than once a day^51^. To simulate multiple applications and ageing periods, our test protocol included the possibility of repeatedly applying toothpastes on the dentin slices. Ageing of teeth between tooth brushing time points was simulated by exposing the specimens to thermal stress.

The ageing procedure applied in the present study was derived from standard thermal ageing protocols^52,53^. We adopted the temperatures (5 °C and 55 °C), as well as the immersion periods (30 s) in the two differently tempered water basins. In the present study one thermal ageing period consisted of 100 cycles, whereas in the former studies on restorations 5000 cycles were applied. We believe three periods of 100 cycles better resembles the thermo-hydraulic stress of the clinical situation when applying a toothpaste on dentin than 5000 cycles.

Considering successive differences of relative hydraulic conductance, *ΔrL*_*p*_ indicates the influence of a specific processing step (material application or thermal ageing, see Table 1), because for each single slice the measurement directly before the processing step was subtracted from the measurement immediately afterwards. Thus, the *ΔrL*_*p*_ values can be considered as the change of hydraulic conductance due to a specific processing step. In this sense, negative *ΔrL*_*p*_ values stand for a decrease, positive for an increase of dentin permeability. A decrease of dentin permeability stands for more occluded dentin tubuli, whereas an increase stands for less occluded tubuli. As expected, in general, material application (t_1_-t_0_, t_3_-t_2_, t_5_-t_4_) resulted in occlusion of dentin tubuli, whereas thermal ageing periods (t_2_-t_1_, t_4_-t_3_, t_6_-t_5_) reopened them to a distinct level (see Table 1 and Fig. 4). Concerning multiple applications and ageing periods, our results clearly showed that the increase of dentin permeability caused by thermal ageing decreased with time (see Fig. 4). This indicates that multiple applications may lead to more occluded dentin tubuli, and thus, provided additional information compared to a single application.

**Table 1:** Sequence of processing steps for a dentin slice including measurements and results.

Measurement	Processing step	Result
	Apply saliva (if applicable)	
	Permeability measurement	*L*_*p*_ (Hydraulic conductance) at t_0_: no material application, no thermal ageing; Baseline
	Apply saliva (if applicable) and material 1	
t_1_	Permeability measurement 1	*L*_*p*_ at t_1_: 1x material, no thermal ageing
	Thermal ageing 1	
t_2_	Permeability measurement 2	*L*_*p*_ at t_2_: 1x material, 1x thermal ageing
	Apply saliva (if applicable) and material 2	
t_3_	Permeability measurement 3	*L*_*p*_ at t_3_: 2x material, 1x thermal ageing
	Thermal ageing 2	
t_4_	Permeability measurement 4	*L*_*p*_ at t_4_: 2x material, 2x thermal ageing
	Apply saliva (if applicable) and material 3	
t_5_	Permeability measurement 5	*L*_*p*_ at t_5_: 3x material, 2x thermal ageing
	Thermal ageing 3	
t_6_	Permeability measurement 6	*L*_*p*_ at t_6_: 3x material, 3x thermal ageing

The saliva collection protocol applied guaranteed the integrity of salivary proteins^54^ and details have been discussed elsewhere^55^. Only one batch of saliva was used for the whole study.

In general, the application of test substances caused a decrease in dentin permeability, except EH without saliva (Fig. 2). The level of the positive control material AL (dashed line) was not reached. A tubuli occluding effect was also found in a clinical study, which reported on about 5% to 48% completely occluded tubules after use of fluoride containing or hydroxyapatite containing tooth pastes twice daily for up to 14 days^56,57^. An earlier clinical study reported a 90% improvement of DH within 3 to 5 days after application of “finely granular hydroxyl apatite” and 90% pain free patients after 4 weeks^58^. The reductions of dentin permeability of BR and SP is probably caused by a layer of deposits on dentin, which can be seen on the SEM pictures without distinct difference of specimens with and without saliva (Figs 6 and 7).

With saliva, EH showed the highest tubuli occluding potential compared to other materials. Without saliva, no reduction was found. This significant influence of saliva on the results of EH can be explained by the fact that the amino acid arginine together with calcium carbonate have been made responsible for the desensitizing effect of saliva. The addition of these substances to the tooth paste EH was meant to enhance this process^42,43,59^. It thus can be assumed that other components of saliva may also be necessary for the dentin permeability reducing effect of EH. Furthermore, in the SEM pictures of EH without saliva, needle like crystals can be seen (Fig. 6), which resemble those of calcium carbonate^60^. These crystals cannot be seen in the SEM pictures when EH was applied with saliva. Again this indicates that saliva may modify components of EH in order to achieve the reduction of dentin permeability^43,59^. Our finding that BR showed highest tubuli occluding potential without saliva has not been reported in the literature.

A general influence of saliva could not be demonstrated in the present study using the Error-Rates-Method. However, without saliva, the hydroxyapatite containing material (BR) showed a higher tubuli occluding effect in terms of a lower area coefficient compared to potassium nitrate (SP) as well as arginine and calcium carbonate (EH) containing materials. In the presence of saliva EH showed the highest effect, and this effect was significantly different compared to without saliva.

Over the course of multiple applications hydraulic conductance of untreated controls increased with time (Fig. 1), which might be explained by a rinsing effect of repeated permeation with water. Multiple applications of toothpastes showed a tendency to decrease hydraulic conductance, whereas ageing cycles by tendency re-opened tubules (Fig. 4). Since there was a tendency for a reduced hydraulic conductance increase after ageing with time, a second and third material application and ageing cycle showed additional information compared to single treatments. Studies on hydraulic conductance of dentin after three applications and three ageing cycles have not been published so far to the best of our knowledge.

A major limitation of the study is the large variation of the data. However, the variations in our study tended to be smaller compared to an alternative study^40^ carried out under similar conditions. We applied a constant and quite low pressure for perfusion of the dentin slices, resulting in largely varying hydraulic conductance values. In the alternative approach the hydraulic conductance of each dentin slice was normalized in advance by applying pressure. Regarding different dentin slices, this pressure is largely varying and quite high. After this normalization, the variation of the resulting hydraulic conductance data seems to be low. Based on these data, with the methods we used in the present study, trends may be detected rather than small differences. Dentin discs with a more standardized and homogeneous hydraulic conductance as test substrate would be desirable and artificially fabricated hydroxyapatite pellets mimicking the dentinal tubular structure could be considered in the future, but may always be challenged as not representing the real situation.

## Methods Test materials and controls

Experimental materials comprised a hydroxyapatite containing toothpaste (BR), a toothpaste containing potassium nitrate (SP), and a toothpaste containing arginine and calcium carbonate (EH). Controls comprised a self-etching adhesive (AL) and an untreated control (UC). Details are summarized in Table 2.

**Table 2:** Test materials and controls used for the permeability measurements with active ingredients applied to the permeated dentin slices.

Brand name (abbreviation)	Allocation (Formulation)	Active substance	Manufacturer	LOT
Biorepair (BR)	Test substance (Toothpaste)	20% zinc hydroxyapatite	Dr. Kurt Wolff GmbH & Co. KG, Bielefeld, Germany	Ref. 80150 PZN: 0985266
Sensodyne Proschmelz (SP)	Test substance (Toothpaste)	Potassium nitrate, sodium fluoride	GlaxoSmithKline Consumer Healthcare GmbH & Co. KG, Bühl, Germany	PZN: 3827214
Elmex Sensitive Professional [home use] (EH)	Test substance (Toothpaste)	Calcium carbonate, arginine, sodium monofluorophosphate	GABA GmbH, Loerrach, Germany	LOT: 019410 PZN: 6810639
Adper Prompt L-Pop (AL)	Control (Self etching adhesive)	Not applicable	3 M Espe AG, Seefeld, Germany	LOT: 370664
No substance (UC)	Control (Not applicable)	Not applicable		

All experimental materials and untreated controls were tested in the presence or absence of human saliva. According to the manufacturer´s instructions the self-etching adhesive (AL) was applied only once and tested without saliva only.

### Dentin slices

Lower permanent incisors of freshly slaughtered, dead bovine animals were used as dentin source. 200 µm thick dentin slices were cut parallel to the tooth axis close to the dental pulp using a low speed saw with an 80-mm radius saw blade at 600 rpm and 0.6 mm s^−1^ crosshead speed under copious water cooling (Innenlochsäge Leitz 1600, Leitz, Wetzlar, Germany). The pulp-facing surfaces were etched for 30 s with 50% (wt/vol) aqueous solution of citric acid, other surfaces were polished^1^ with ANSI 600 grit SiC paper for 10 s at 100 rpm and application of 2.5 N pressure under copious water cooling (Metaserv Motopol 8, Buehler, UK).

### Saliva

Human saliva was obtained from two co-authors who performed the experiments (IG, CN) after verbal informed consent. Approval of an ethics committee is not required, since potentially ethically relevant issues were not affected as confirmed by our ethics committee (Ethikkommission, Universität Regensburg). Both authors voluntarily used their own saliva according to the study protocol, whereby any potential risks of health hazards by using saliva from foreign donors could be excluded. Saliva was only used for precoating dentin slices and no analyses were performed. Saliva donors were non-smoking and healthy. Saliva was filtered under sterile conditions as developed in our laboratory and described in detail^54^. About 150 ml of pooled whole saliva was collected once for the whole study from the two donors. Before expectorating, donors did neither eat nor drink for 30 min and cleaned their mouth using water. A piece of a paraffin-polyethylene foil (parafilm; Pechiney Plastic Packaging Company, Chicago, IL) was used to stimulate saliva flow. Saliva was expectorated, collected in sterile tubes (Blue Max, Falcon; Becton Dickinson Labware, Franklin Lakes, NJ), stored on ice and finally pooled from the two donors. Saliva then was repeatedly drawn through a sterile needle (0.9 by 22 mm; Miraject, Duisburg, Germany) fixed on a sterile syringe (5 ml, Luer-Lok; Becton Dickinson, Drogheda, Ireland), in order to decrease viscosity. Afterwards, saliva was sterile filtered in a laminar flow hood by passing it consecutively through sterile syringe filters of decreasing pore size (Minisart CE single-use syringe filters, hydrophilic; ranging from 5.0 to 0.2 µm; Sartorius AG, Göttingen, Germany). Samples of 2 ml each were stored at −20 °C.

### Material application

Polished non pulp-facing surfaces of dentin slices were gently dried using a soft paper towel and, if applicable, 5 µl human saliva was spread out on the permeation area and a pea-sized amount of test material was moderately brushed during 15 s onto this surface (Brush Tips, White Shank, 10 mm, size M). Excessive material was removed with 30 ml aqua dest. using a syringe (BD Plastipak Perfusion Syringe 50 ml).

The self-etching adhesive control material AL was applied according to the manufacturers´ instructions. The not pulp-facing surfaces of dentin slices were gently dried using a soft paper towel. AL was applied for 15 s using the applicator provided, thereby rubbing the solution with moderate pressure onto the dentin surface. A gentle stream of air was used to thoroughly dry the adhesive to a thin film, which then was light cured (ESPE Elipar Trilight (460 mW, corresponding to a light intensity of 830 mW cm^−2^)) for 10 s. A second layer of adhesive material was applied and light cured accordingly.

Untreated control slices (UC), without material application, were gently dried as described above. If applicable, saliva was applied, but no further material was placed onto the surfaces.

### Hydraulic conductance and Experimental timeline

**Figure 9:**
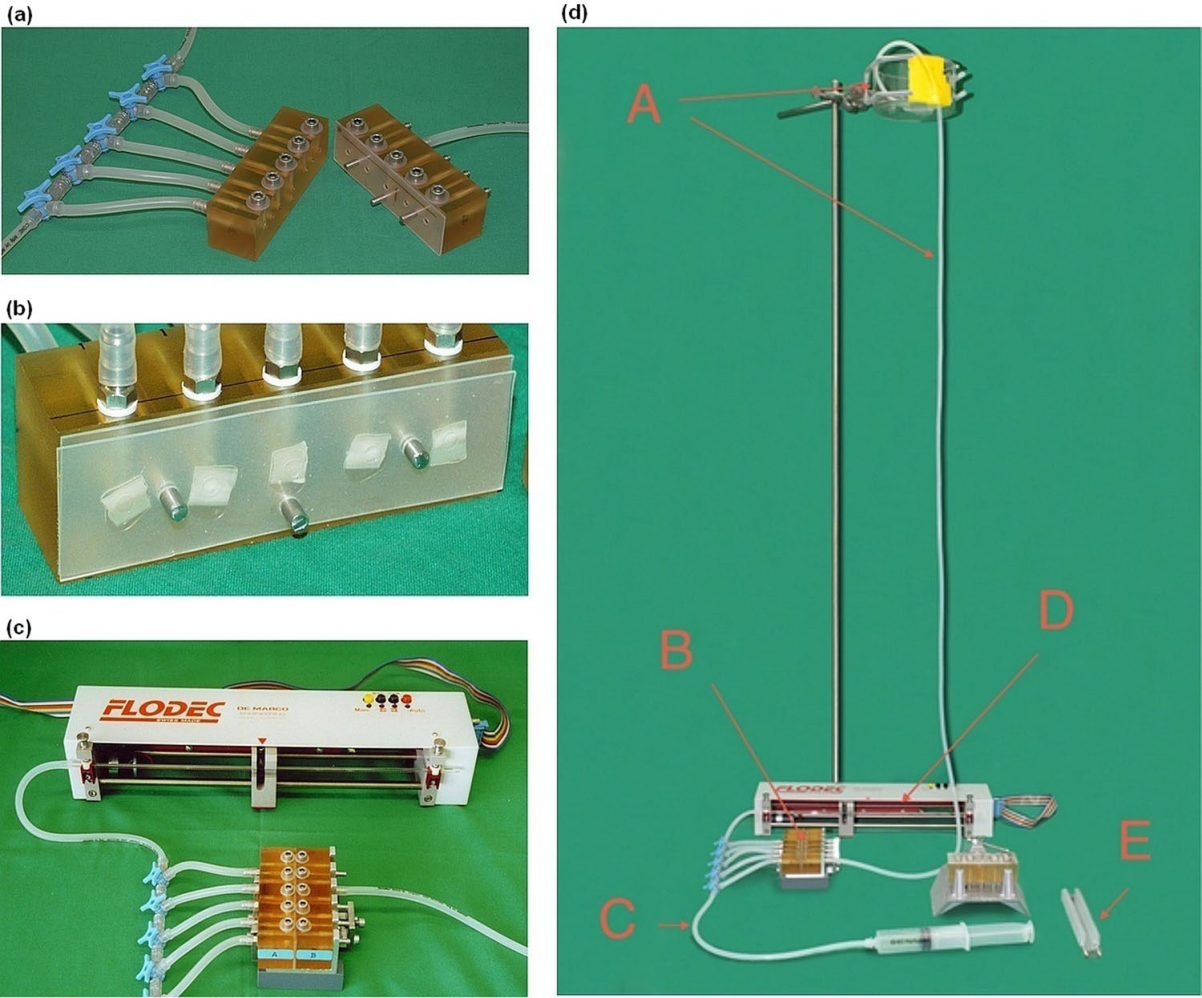
Details of the experimental setup. (**a**) open permeability chamber, facing surfaces are covered with a silicone mat to hold five dentin slices. (**b**) detailed view of dentin slices within the silicone mats on one part of the permeability chamber. (**c**) Flodec apparatus connected to the closed permeability chamber. (**d**) complete experimental setup. (**A**) water column with reservoir; (**B**) closed permeability chamber; (**C**) tube for deaeration; (**D**) Flodec measuring unit including glass capillary with a constant inner diameter of 873 µm (details see Fig. 10); (**E**) dentin slice holder for thermal ageing. Copyright of the Flodec logo given to Macmillan Publishers Ltd, part of Springer Nature.

**Figure 10:**
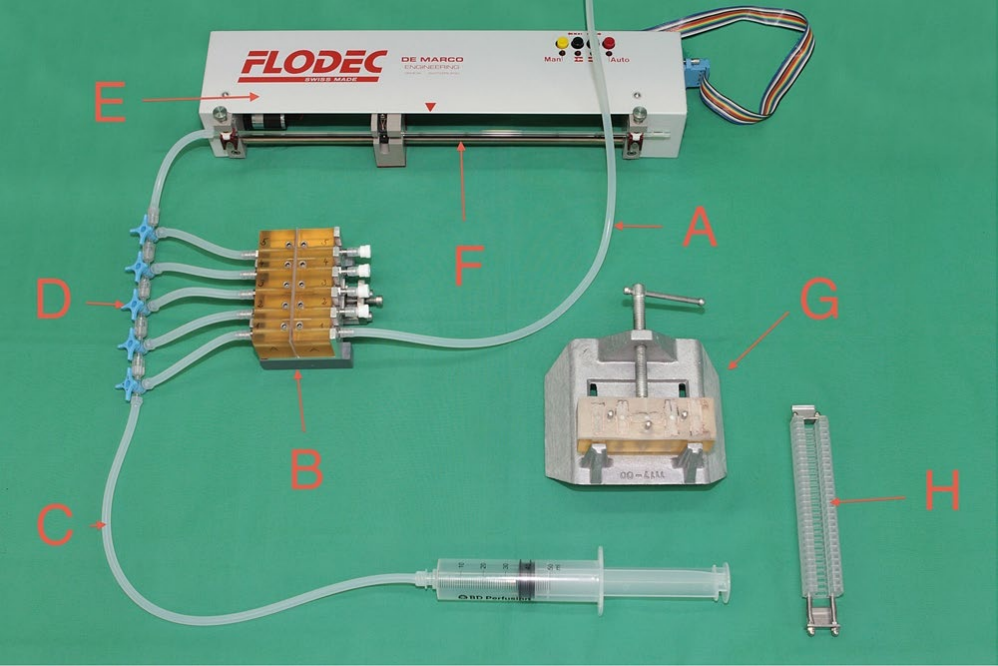
Flodec measuring unit in detail. The glass capillary (**F**) as a part of the unit and the closed permeability chamber (**B**; details see Fig. 9) are connected with a tube, and a dentin slice, which is inserted into the permeability chamber (**B**) may be permeated by opening the appropriate 3-way valve (**D**). Tap (**C**) and connected syringe are used to deareate the system. Tap (**A**) is the lower part of the water column (see Fig. 9(d)). Apparatus (**G**) is a custom made holder for material or saliva application onto dentin slices, and (**H**) shows the dentin slice holder for thermal ageing. Copyright of the Flodec logo given to Macmillan Publishers Ltd, part of Springer Nature.

Dentin permeability was evaluated as hydraulic conductance (*L*_*p*_, m^3^ N^−1^ s^−1^) using aqua dest. at a pressure of 1.4 m H_2_O column using a commercially available measurement unit (Flodec, DeMarco-Engineering SA, Switzerland) (Figs 9 and 10) at room temperature. A dentin slice was placed in a flow chamber, sealed by silicone plates in order to allow aqua dest. to flow only through dentin tubules of a circular area of 12.6 mm^2^. Fluid flow through the glass capillary with a constant inner diameter of 873 µm was recorded during 120 s and hydraulic conductance was calculated as described below (Data treatment and statistical analyses).

During hydraulic conductance measurements the dentin slices were fixed in the permeability chamber (Fig. 9). For thermal ageing, slices were removed from the permeability chamber and immediately put into a custom made holder (Figs 9(d),E and 10,H), which was mounted into the apparatus for thermal ageing. One cycle of thermal ageing consisted of immersing the dentin slices successively in two baths containing water at 5 °C and 55 °C for 30 s each with a transfer time of 5 s. One period of thermal ageing consisted of 100 cycles^52,61^. In case of a repeated hydraulic conductance measurement, the slices were placed back into the permeability chamber in an identical position. The slices were kept under wet conditions at room temperature, from cutting until the end of their last measurement, except for the thermal ageing periods (wet, but not room temperature).

Permeability of a dentin slice was measured at baseline (t_0_), then after a first material application (t_1_), and again after a first thermal ageing period (t_2_). Permeability measurements after two more cycles of material application (t_3_, t_5_) and thermal ageing periods (t_4_, t_6_) followed (Table 1).

### Scanning electron microscopy (SEM)

Exemplarily, SEM images (FEI Quanta 400 F, FEI Europe, Eindhoven, NL) of dentin slices after treatments (BR, SP, EH) without and with saliva application were taken of the dentin surface at magnifications of 5,000x and 20,000x under environmental conditions. Specimens were prepared as described above and removed from the experiment after material application (t_1_) or the first period of thermal ageing (t_2_), respectively. Accordingly, SEM images were taken at t_1_ and t_6_ of untreated controls. Specimens were kept at 100% relative humidity for at most 24 h at 7 °C before visualization without any further preparation under low vacuum conditions (1.5 Torr) using a large field detector including a pressure limiting aperture at a working distance of 10 mm and an acceleration voltage of 4 kV with a field emitter.

### Data treatment and statistical analyses

Using the Flodec apparatus fluid flow through each dentin slice was continuously recorded for 120 s. The first and last 15 s were discarded, and the resulting values of the inner 90 s measurement period were graphically visualized in a time-traverse diagram and fitted (TableCurve 2D V 5.01, SYSTAT Software Inc., SanJose, CA, USA). Data were submitted into a linear fit with Pearson´s correlation coefficient r^2^ being the measure for the quality of the fit (Fig. 8). The resulting slope [mm s^−1^] of the fitted straight line was considered representative for the fluid flow through the dentin slice. Since the inner diameter of the glass capillary was constant over the whole length (information of the manufacturer), this slope could be directly transformed into a fluid flow, expressed in the unit [ml s^−1^]. Using these fluid flow values and the pressure applied, *hydraulic conductance L*_*p*_ [m^3^N^−1^s^−1^] of the dentin slice considered was calculated for each of the seven measurements. For each dentin slice the six *L*_*p*_ values (at t_1_, t_2_,…. t_6_) after their baseline (t_0_) measurement were related to their baseline value and expressed as percentage *rL*_*p*_ (100% = *L*_*p*_ at baseline, %)

1 $$r{L}_{p}(at\,{t}_{i})=\,\frac{{L}_{p}\,(at\,{t}_{i})}{{L}_{p}\,(at\,{t}_{0})}\ast 100\,[ \% ]\,(i=1,2,\ldots 6)$$

(Table 1). In this sense, each dentin slice served as its own control. *Difference of relative hydraulic conductance*

2 $$\Delta r{L}_{p}=r{L}_{p}(at\,{t}_{n})-r{L}_{p}(at\,{t}_{n-1})(n=1,2,\ldots 6)$$

as successive differences of *rL*_*p*_ between time points t_n_ and t_n−1_ was calculated to visualize directly the influence of material application (t_1_-t_0_, t_3_-t_2_, t_5_-t_4_) or thermal ageing (t_2_-t_1_, t_4_-t_3_, t_6_-t_5_) (Table 1).

To gain an overview of the results, for each slice the area under it´s *rL*_*p*_ curve (abscissa: measurement time; ordinate: corresponding *rL*_*p*_ value) was calculated, related to matching areas under the curves of the untreated controls (UC), and expressed as percentage (*area coefficient*, %; 100% ~ area under the curves of untreated controls)

3 $$area\,coefficient\,(of\,specific\,slice)=\frac{\,area\,of\,the\,r{L}_{p}\,curve\,of\,specific\,slice}{area\,of\,the\,r{L}_{p}\,curves\,of\,the\,matching\,untreated\,controls}\ast 100\,[ \% ]$$

The hydraulic conductance of a dentin slice was measured seven times (t_0_, t_1_, …, t_6_; Table 1) during one workday. A dentin slice was accepted for analysis, if all seven corresponding correlation coefficients r^2^ of the linear fits were 0.7 or higher. At least 10 samples per experimental or control group were used. Data were depicted as medians including 25–75% quantiles. Mann-Whitney-U-Test and Wilcoxon Test were used for statistical analysis (0.05 level of significance) of independent and dependent values, respectively (SPSS Statistics V23, SPSS Inc. Chicago, USA). The Error-Rates-Method was applied to generally test the influence of parameters^62^.

## Conclusion

Generally, all toothpastes tested were able to reduce dentin permeability, except EH without saliva. In this case the Null hypothesis was rejected. The hydroxyapatite containing toothpaste reduced dentin permeability significantly more in absence of saliva, whereas in presence of saliva the arginine and calcium carbonate containing product was superior compared to the HAP toothpaste. However, due to the lack of significance in these cases, the Null hypothesis could not be rejected. Results from EH were in line with the literature, whereas results from BR were newly reported. The newly introduced test conditions influenced single results significantly and as they better simulate the *in vivo* situation they should be considered to be included in further *in vitro* permeability testing of desensitizing preparations.

### Data Availability Statement

All relevant data are within the paper files.
